# Minoxidil Beyond Hair: A Comprehensive Review of Its Effects on Nail Growth

**DOI:** 10.3390/jcm15051792

**Published:** 2026-02-27

**Authors:** Víctor Pinos-León, Jorge Vasconez-Gonzalez, Juan S. Izquierdo-Condoy, Belén Pazmiño, Jenny Sánchez, Vianca Torres, Esteban Ortiz-Prado

**Affiliations:** 1 Grupo Derma AID, Quito 170103, Ecuador; 2 Hospital Metropolitano de la Ciudad de Quito, Quito 170509, Ecuador; 3 One Health Research Group, Faculty of Health Science, Universidad de Las Americas, Ecuador Calle de los Colimes y Avenida De los Granados, Quito 170137, Ecuador

**Keywords:** minoxidil, nail growth, topical formulation, oral minoxidil, vascular endothelial growth factor, Wnt signaling

## Abstract

Minoxidil is a well-established treatment for hair loss, known for its vasodilatory properties and effects on dermal papilla cells. Recently, interest has emerged regarding its potential role in promoting nail growth, both in healthy individuals and in those with nail dystrophies. The objective of this review was to synthesize and critically analyze the available evidence on the effects of topical and oral minoxidil on nail growth, including proposed mechanisms of action, clinical outcomes, and safety. A narrative literature review was conducted using studies published in indexed journals that evaluated the pharmacological effects of minoxidil on the nail unit. Mechanistic data were extracted from in vitro and in vivo models, while clinical results were compiled from pilot studies, case reports, and trials evaluating nail growth in both healthy and pathologic conditions. Proposed mechanisms include vasodilation, increased VEGF expression, and hypothesized involvement of pro-growth pathways (e.g., Wnt/β-catenin), largely extrapolated from hair follicle biology; direct nail-matrix-specific mechanistic evidence remains limited. Clinical studies with 5% topical minoxidil have shown increases in nail growth rate ranging from 13% to 19% over 28 days, while oral minoxidil (2.5 mg) resulted in an approximately 50% increase in nail growth speed in small studies. Evidence also suggests improvements in nail strength and appearance. Adverse effects were minimal, with no significant changes in blood pressure and only modest increases in heart rate with oral formulations. Although evidence remains limited, preliminary findings suggest a potential role for minoxidil in enhancing nail growth, particularly in cases of slowed growth or nail dystrophy. More robust, large-scale studies are needed to confirm efficacy, determine optimal formulations, and establish long-term safety. Given its accessibility and tolerability, minoxidil may represent a promising off-label adjunct for nail growth modulation.

## 1. Introduction

The basic anatomy of the nail unit consists of the nail plate, nail matrix, nail bed, nail folds, and hyponychium [1]. It has abundant arterial irrigation through the pairs of dorsal digital arteries, and venous drainage via the superficial and deep systems. Additionally, it is innervated by the dorsal branches of the digital nerves [2].

Regarding the growth rate, it is typically around 3 mm per month for fingernails and 1 mm per month for toenails. This tends to decrease starting at the age of 25, at a rate of 0.5% per year [3,4]. In total, the growth of a fingernail takes between 4 to 6 months, and a toenail between 12 to 18 months. This growth rate depends on the ability of the metabolically active cells in the matrix to proliferate and may increase or decrease due to physiological, pathological, or medicinal conditions [5]. Factors that influence the decrease in growth include advanced age, anatomical location, extracutaneous disorders such as fever, malnutrition, infections, immobilization, skin disorders like onychomycosis, and drugs such as L-DOPA, azathioprine, methotrexate, and etretinate [6]. Diseases characterized by this alteration include yellow nail syndrome, onychomycosis, lichen planus, and brittle nail syndrome, in which there is nail dystrophy, and the patient tends to experience a negative impact related to their self-esteem [5].

While it is well known that treatment is directed at the etiology of these diseases, it is often difficult to manage in certain situations. Therefore, it is inferred that the use of growth enhancers, such as minoxidil, may help and positively influence the growth of both normal and dystrophic nails [7]. Moreover, this drug is the subject of study because it is easily accessible, low-cost, and has been reported to have few adverse effects [8].

Minoxidil, known as 2,4-pyrimidinediamine, 6-(1-piperidinyl)-3-oxide, was primarily used as an oral antihypertensive drug due to its peripheral vasodilatory effect. However, in 1987, a topical formulation was developed due to a reported side effect in men, namely hypertrichosis [9]. Although it was only approved by the FDA in 1988 as a treatment for androgenetic alopecia, it has been proposed that its mechanism of action on the hair follicle may work similarly to its effect on the nail unit, due to the structural similarities and its potential impact on periungual blood flow [8,10].

Due to the limited available information, a study has been developed to synthesize the evidence and highlight key points detailing the benefits of both oral and topical minoxidil on nail growth. This will allow for a better understanding of the topic, which could be useful in clinical decision-making and serve as an incentive for future research.

## 2. Search Strategies

**Figure 1:**
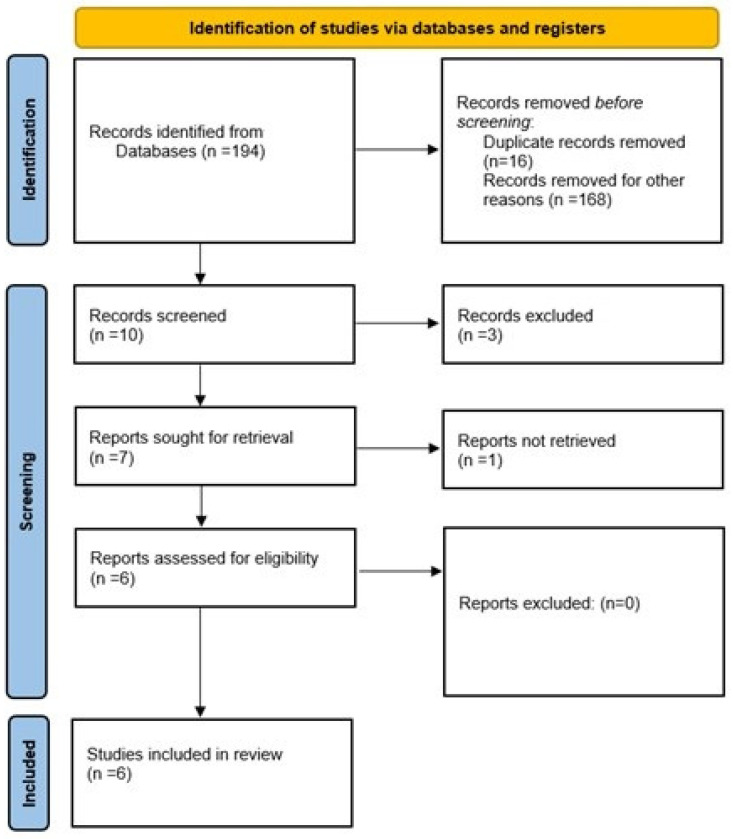
Study Selection Process Following PRISMA Guidelines. PRISMA flow diagram showing the number of records identified, screened, assessed for eligibility, and included in the review.

For the development of this narrative review, original studies were included, including case reports and case series. Opinion articles, perspectives, editorials, letters to the editor, systematic reviews, and meta-analyses were excluded. Articles were retrieved from PubMed, Scopus, and Web of Science databases. The search was conducted in both English and Spanish, without restrictions on the year of publication. The following search strategy was used ((“minoxidil” OR “oral minoxidil” OR “topical minoxidil”) AND (“nails” OR “nail growth” OR “nail disorders”)). After the review process, six articles were included (Figure 1).

## 3. Nail Biology and Growth Physiology

The maintenance of nail growth is due to two autonomous populations of skin stem cells: the fast-cycling, high-proliferation stem cells in the nail matrix, which replenish the nail plate [11]. Meanwhile, the slow-cycling cells in the proximal nail fold contribute to the periungual epidermis under normal homeostasis conditions and to the nail structure after injury [11].

Two signaling pathways that contribute to nail growth have been identified. Bone morphogenetic protein (BMP) signaling has been shown to require the downstream transcription factors of BMP, known as Msx2 and Foxn1, for the proper terminal differentiation of onychocytes and the organization of the underlying nail bed and the eosinophilic keratogenous zone. This signaling pathway instructs the stem cells of the nail mini-organ to differentiate into the nail plate [11].

On the other hand, the Wnt signaling pathway has been observed to regulate the development of nail organs in embryos and is essential for the formation of the nail plate. Studies in animals have shown that deactivating the Wnt pathway leads to an expansion of the proximal matrix and the replacement of the hard nail structure with sections of highly proliferative nail epithelium [11].

## 4. Therapeutic Rationale for Minoxidil Use in Nails

Minoxidil has long been recognized for its efficacy in treating androgenetic alopecia due to its vasodilatory and proliferative effects on hair follicle cells [12]. Given the structural and functional similarities between the hair follicle and nail matrix, both being sites of active keratinocyte proliferation, its use has been proposed as a potential enhancer of nail growth. Furthermore, its accessibility, favorable safety profile, and low cost make it an attractive off-label option for conditions involving slowed or altered nail growth, such as yellow nail syndrome or brittle nail syndrome. This section outlines the clinical reasoning and biological plausibility supporting its application in nail disorders.

### 4.1. Mechanisms of Action: From Vasodilation to Wnt Signaling

**Figure 2:**
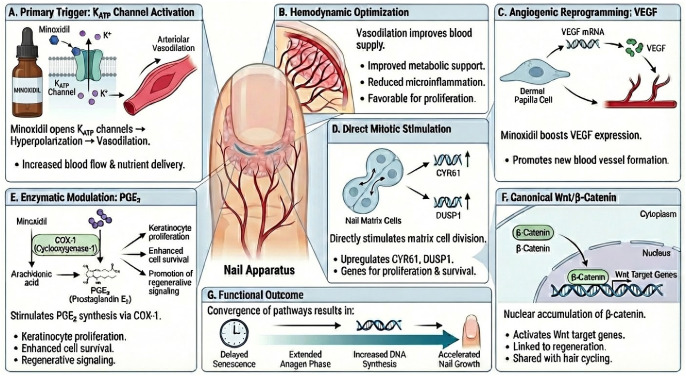
Multifactorial molecular mechanisms of Minoxidil in the nail apparatus. The diagram illustrates the synergistic pathways through which minoxidil promotes nail growth. (**A**) Activation of ATP-sensitive potassium (KATP) channels in vascular smooth muscle induces hyperpolarization and arteriolar vasodilation. (**B**) This hemodynamic optimization enhances oxygen and nutrient supply to the nail matrix, reducing local microinflammation. (**C**) At the molecular level, the drug upregulates vascular endothelial growth factor (VEGF) mRNA expression, fostering an angiogenic environment. (**D**) Simultaneously, direct mitotic stimulation of matrix cells occurs via the upregulation of key proliferative genes such as *CYR61* and *DUSP1*, a mechanism independent of blood flow. (**E**) Enzymatic modulation via cyclooxygenase-1 (COX-1) increases Prostaglandin E2 (PGE2) synthesis, favoring keratinocyte proliferation. (**F**) Activation of the canonical Wnt/β-catenin pathway promotes nuclear accumulation of β-catenin, activating target genes related to tissue regeneration. (**G**) The convergence of these pathways results in delayed cellular senescence and an extension of the anagen phase, leading to accelerated and sustained nail growth.

Minoxidil acts primarily as a potassium channel opener, activating ATP-sensitive K+ (KATP) channels in vascular smooth muscle and inducing membrane hyperpolarization (Figure 2A) [13,14]. This triggers arteriolar vasodilation and improves local perfusion, thereby enhancing oxygen and nutrient delivery to highly proliferative keratinized tissues, including the nail matrix (Figure 2B) [13,14]. In parallel, minoxidil has been associated with immunomodulatory effects, including reduced release of pro-inflammatory mediators (e.g., interleukin-1α) and attenuation of local immune activity, which may mitigate microinflammation in the periappendageal environment (Figure 2B) [13].

Beyond hemodynamic effects, minoxidil appears to influence epithelial cell biology and regenerative programs. Experimental data in hair models indicate increased VEGF expression and release in dermal papilla cells [13,15]; by extension, within the nail apparatus, minoxidil has been hypothesized to promote an angiogenic milieu through upregulation of VEGF mRNA expression (Figure 2C) [8]. Minoxidil has also been linked to prostaglandin pathway modulation, including increased prostaglandin E2 synthesis via cyclooxygenase-1 (COX-1), which may favor keratinocyte proliferation and survival (Figure 2E) [16]. In vitro, nuclear accumulation of β-catenin and increased transcription of Wnt target genes have been observed after drug exposure [14], consistent with engagement of canonical Wnt/β-catenin signaling implicated in keratinized tissue regeneration (Figure 2F). Additionally, nail-related transcriptomic signals suggest that minoxidil may directly stimulate nail-matrix cell proliferation through upregulation of CYR61 and DUSP1, representing a mitogenic effect that may occur independently of blood-flow changes (Figure 2D) [8].

Collectively, the convergence of these vascular, enzymatic, and regenerative pathways provides biological plausibility for accelerated and sustained nail growth under minoxidil exposure (Figure 2G) [16]. Thus, shared vascular (VEGF) and regenerative (Wnt/β-catenin) mechanisms, together with proposed nail-matrix gene induction (CYR61/DUSP1), support minoxidil as an off-label enhancer of nail growth (Figure 2) [13,16].

### 4.2. Pharmacological Profile: Pharmacokinetics and Pharmacodynamics

Minoxidil, a potent vasodilator primarily used for treating hypertension and androgenetic alopecia, demonstrates rapid oral absorption in healthy individuals, achieving peak plasma concentrations within an hour and exhibiting a plasma half-life of approximately 3–4 h [17,18]. The drug undergoes extensive hepatic metabolism, predominantly through glucuronidation, with about 97% of the administered dose recovered in urine, mainly as metabolites, including a glucuronide conjugate, while only 1.4% of topically applied minoxidil is absorbed through the skin [13,19]. Its antihypertensive effects persist longer than its plasma concentrations, likely due to prolonged action at vascular receptor sites [17,20]. For androgenetic alopecia, topical minoxidil increases cutaneous blood flow in balding scalps, with 5% formulations showing superior efficacy compared to 2% solutions, and low-dose oral minoxidil also proving effective for hair growth [13,21]. Patients with hepatic impairment may require dosage adjustments due to altered pharmacokinetics, as highlighted by Adams et al., emphasizing the need for individualized dosing regimens based on the severity of hypertension or hepatic function [19].

### 4.3. Clinical Evidence of Nail Growth Enhancement

It increases cutaneous blood flow through an enhanced expression of the vascular endothelial growth factor generated by minoxidil, which could have positive effects on the nail growth rate in both normal and dystrophic nails [7]. It has also been mentioned that nail growth could be due to mitotic stimuli directly on the nail-matrix cells through the overregulation of the CYR61 and DUSP1 genes [8].

Benefits of topical minoxidil use on nail growth have been observed; a study conducted in 2017 with 32 patients, who were instructed to use two squirts of a 5% topical minoxidil solution twice daily on two fingernails at the proximal nail fold, revealed that from the first week, the average length of the treated nails was greater than that of the nails in the untreated group. During the first month, the average growth of the treated nails was 4.27 mm/month, compared to 3.91 mm/month in the untreated nails (*p* = 0.003); however, no difference in the nail growth rate was observed after the second month [10]. On the other hand, Garbers et al. evaluated the efficacy of 5% topical minoxidil and 2.5 mg of oral biotin to increase nail growth rate (NGR) in healthy adults over 28 days. The results revealed that the group treated with minoxidil alone showed a 19% increase in the nail growth rate, while the groups receiving biotin and the combination of minoxidil and biotin showed an increase of 13% and 14%, respectively [8]. After 28 days, minoxidil had a higher growth rate than biotin (*p* < 0.01). It was also reported that individuals who bit their nails had a higher nail growth rate (*p* < 0.01) [8] (Table 1).

Starece et al. in their study revealed that the daily application of a drop of 5% minoxidil solution to the proximal nail fold of toenails with growth arrest or onychomadesis produced a clinical response rate of 36% and 81% after 6 and 12 months, respectively [22].

Regarding the oral use of minoxidil, a study evaluating the effect of oral minoxidil at 1 mg and 2.5 mg on nail growth rate revealed that the 2.5 mg dose increased the nail growth rate by 50.7% (*p* < 0.01), while the 1 mg dose did not produce any changes [23]. It has also been reported that oral minoxidil increases patient satisfaction regarding nail strength, faster growth, and a more pleasant appearance [24].

Algain reports the case of a 66-year-old patient with yellow nail syndrome, who upon physical examination presented yellow, brittle nails with slow growth that did not respond to fluconazole and vitamin E. However, the patient was later treated with oral terbinafine and prescribed 2% topical minoxidil. After 6 months, complete remission was observed in all nails, except for the toenails. During a 72-month follow-up, the patient did not experience recurrence of the nail lesions [25].

**Table 1:** Summary of studies evaluating the effects of topical and oral minoxidil on nail growth. This table summarizes the main characteristics of the studies included in this narrative review assessing the effects of topical and oral minoxidil on nail growth. Information regarding study design, population, dosage, clinical outcomes, and reported adverse effects is presented when available.

Author	Study Type	Population	Dose	Outcome	Adverse Effects
Garber et al., 2021 [8]	Quasi-experimental, open, controlled study with a factorial design	Healthy participants	Topical 5% minoxidil spray applied twice daily, and oral biotin 2.5 mg once daily for 28 days.	After 28 days, minoxidil had a higher growth rate than biotin (*p* < 0.01)	No adverse effects were reported
Aiempanakit et al., 2017 [10]	Clinical Trial	Healthy participants	Two squirts of a 5% concentration of topical minoxidil solution twice daily from two months	During the first month, the mean growth of the treated nails was 4.27 mm/month, compared with 3.91 mm/month in the untreated nails (*p* = 0.003).	No adverse effects were reported
Algain 2021 [25]	Case report	66-year-old woman with Yellow Nail Syndrome	Topical 2% minoxidil	After 6 months, complete remission was observed in all nails, except for the toenails	No adverse effects were reported
Alsalhi et al., 2023 [24]	Cross-sectional analysis	Healthy patients	Oral minoxidil (0.25–5 mg daily)	After starting oral minoxidil, 53.0% reported their nails grew more quickly, 37.9% reported their nails became stronger, and 36.4% reported their nails looked nicer.	The authors did not report the presence or absence of adverse effects.
Barbosa et al., 2024 [23]	Open study	Healthy patients	Oral minoxidil 1 mg/day, followed by 2.5 mg/day for 14 days	Minoxidil 2.5 mg, but not 1 mg, increased nail growth speed by 50.7% (*p* < 0.01)	Increased heart rate
Starace et al., 2023 [22]	Retrospective study	Arrest of nail growth or onychomadesis in 50 patients	Daily application of one drop of 5% minoxidil solution to the proximal nail fold of the toenails.	A clinical response rate of 36% and 81% after 6 and 12 months, respectively.	Information not available.

### 4.4. Safety and Adverse Effects in Nail Applications

During topical minoxidil treatment for nails, no skin-related side effects have been reported, nor were there any changes in blood pressure or pulse rate. Regarding oral minoxidil treatment, it has been reported that the heart rate increased by 9.7% with a 1 mg dose and by 16.7% with a 2.5 mg dose (*p* < 0.01), while no changes in blood pressure were reported [10,23].

## 5. Expert Opinion: Clinical and Public Health Perspectives

We believe that the emerging role of minoxidil in addressing unmet needs in nail health—particularly in conditions characterized by impaired growth and structural abnormalities such as yellow nail syndrome, onychomycosis, and brittle nail syndrome—is both promising and clinically relevant. The evidence synthesized in our review demonstrates that 5% topical minoxidil can increase nail growth by 13–19% over 28 days [8,10], while 2.5 mg of oral minoxidil may enhance growth by over 50% [23]. These outcomes are biologically plausible, considering minoxidil’s known vasodilatory action, its ability to upregulate VEGF, and its proposed stimulation of the Wnt/β-catenin signaling pathway—mechanisms that are likely shared between hair follicle and nail-matrix cells.

From a clinical standpoint, minoxidil stands out for its accessibility, affordability, and overall favorable safety profile. Topical formulations are well tolerated, and oral use—while associated with mild increases in heart rate—has not shown significant blood pressure alterations in the studies reviewed [10,23]. The case report of a 66-year-old patient with refractory yellow nail syndrome achieving near-complete remission using 2% topical minoxidil and oral terbinafine [25] exemplifies its potential in difficult clinical scenarios. Furthermore, patient-reported improvements in nail strength, aesthetics, and satisfaction with oral minoxidil [24] suggest that its benefits may extend beyond mere acceleration of growth, contributing to quality of life.

However, this promise must be interpreted with caution. The evidence remains preliminary derived primarily from small, short-term trials and retrospective case reports, with considerable methodological variability. For instance, studies such as [8,10] provide compelling early data but lack the sample size, duration, and standardized outcome measures necessary for definitive clinical guidance. Mechanistic hypotheses, while reasonable, still rely heavily on extrapolations from follicular models, and nail-specific cellular responses remain validated.

As a physician-scientist engaged in both dermatological practice and public health research, I see in minoxidil a unique opportunity to bridge therapeutic innovation and health equity. In low- and middle-income countries such as Ecuador, where access to advanced dermatological therapies is often limited, minoxidil’s low cost and over-the-counter availability position it as a feasible, scalable solution for common and often neglected nail disorders. From a cost–benefit perspective, especially in resource-constrained settings, minoxidil could offer substantial value—not only by improving individual outcomes but by reducing the long-term burden of untreated or refractory nail conditions.

Looking ahead, I strongly advocate for rigorous, multicenter randomized controlled trials to confirm its efficacy, determine optimal dosing regimens, and evaluate long-term safety. Additionally, research exploring combination protocols—such as pairing minoxidil with antifungals, biotin, or anti-inflammatories—could further enhance its therapeutic profile. Additionally, randomized controlled clinical trials should be conducted to analyze the effect of minoxidil on specific nail disorders, such as yellow nail syndrome, onycholysis, and nail psoriasis, with the aim of adequately evaluating the efficacy and safety of topical and oral minoxidil in this context. Until such evidence is available, off-label use should be pursued thoughtfully, ensuring appropriate patient selection, counseling, and monitoring.

In summary, minoxidil may represent a cost-effective and clinically meaningful tool for nail regeneration, with the potential to redefine treatment paradigms—particularly in contexts where conventional therapies are either inaccessible or insufficient.

## 6. Limitations

This review is subject to several limitations that must be acknowledged. First, the current evidence based on the effects of minoxidil on nail growth is limited in both quantity and quality. Most of the available studies are small-scale, non-randomized, and lack robust methodological designs. For instance, the principal clinical findings are derived from pilot studies, retrospective analyses, or single case reports, which restrict the generalizability and external validity of the results.

Second, the duration of follow-up in most studies is short (typically 28 days to a few months), which limits our understanding of the long-term efficacy and safety of minoxidil for nail-related indications. Adverse effects, especially with prolonged oral administration, remain underreported and require further investigation.

Third, mechanistic insights into how minoxidil stimulates nail growth are largely extrapolated from studies on hair follicles and dermal papilla cells. While the proposed pathways—such as VEGF upregulation and Wnt/β-catenin activation—are biologically plausible, there is insufficient direct evidence from nail-matrix cell studies to confirm these mechanisms in nail-specific contexts.

Fourth, there is significant heterogeneity in dosing regimens, treatment durations, patient populations, and outcome measurements across the reviewed studies. This variability complicates direct comparisons and precludes meta-analytic synthesis or standardized clinical recommendations.

Finally, publication bias may also be present, as studies reporting positive outcomes are more likely to be published, potentially overestimating the true efficacy of minoxidil in nail growth promotion.

## 7. Future Directions in Research and Clinical Practice

To overcome current knowledge gaps, future research should prioritize the design and execution of large-scale, multicenter randomized controlled trials with rigorous methodology, standardized treatment protocols, and adequate follow-up durations. These studies should evaluate both efficacy and safety across diverse populations and clinical settings. Additionally, dedicated mechanistic investigations focusing specifically on nail-matrix biology are needed to validate the proposed molecular pathways—such as VEGF upregulation and Wnt/β-catenin activation—in the context of nail growth. Generating high-quality evidence through such studies is essential to develop clear, evidence-based clinical guidelines for the safe, effective, and targeted use of minoxidil in the management of nail disorders.

## 8. Conclusions

The evidence regarding the benefits of minoxidil for nail growth remains limited. Available data suggests that 5% topical minoxidil may modestly increase nail growth rate in small studies, while oral minoxidil at 2.5 mg has been associated with larger increases in nail growth speed in limited observational data. However, the current literature is sparse and heterogeneous, and there are no robust head-to-head comparisons; therefore, definitive conclusions regarding the superiority of topical versus oral regimens cannot be made. The lack of large-scale studies also limits generalizability. Further research, including adequately powered randomized trials with standardized outcome measures, is required to confirm efficacy, clarify mechanisms relevant to the nail unit, identify the clinical contexts in which therapy is most beneficial, and establish short- and long-term safety. Future work should also define appropriate dosing and duration, develop guidance for use across different nail disorders, and explore optimized formulations or combination strategies to enhance therapeutic effect.

## Data Availability

Not applicable.

## Abbreviations

The following abbreviations are used in this manuscript:

BMP	Bone morphogenetic protein
VEGF	vascular endothelial growth factors
NGR	nail growth rate
